# Detecting *Dirofilaria immitis*: Current Practices and Novel Diagnostic Methods

**DOI:** 10.3390/pathogens13110950

**Published:** 2024-10-31

**Authors:** Damian Pietrzak, Julia Weronika Łuczak, Marcin Wiśniewski

**Affiliations:** 1 Division of Parasitology and Parasitic Diseases, Department of Preclinical Sciences, Institute of Veterinary Medicine, Warsaw University of Life Sciences, 02-786 Warsaw, Poland; damianpietrzak23@gmail.com; 2 Department of Molecular Biology, Institute of Genetics and Animal Biotechnology, Polish Academy of Sciences, Postępu 36A, 05-552 Magdalenka, Poland; j.luczak@igbzpan.pl; 3 Department of Nanobiotechnology, Institute of Biology, Warsaw University of Life Sciences, 02-786 Warsaw, Poland

**Keywords:** *Dirofilaria immitis*, nematoda, dirofilariosis, microscopy-based methods, diagnostics, serological test, molecular biology techniques

## Abstract

The nematode *Dirofilaria immitis* is responsible for a vector-borne disease affecting canines and humans worldwide, known as cardiopulmonary dirofilariasis. An accurate and early diagnosis is of the utmost importance for effective disease management. While traditional microscopy-based methods remain invaluable, they have inherent limitations. Serological tests, in particular ELISA and immunochromatographic tests, are employed due to their capacity to detect *D. immitis* antigens, offering ease of use and diagnostic accuracy. The advent of molecular methods has the potential to enhance routine diagnostic approaches, with polymerase chain reaction (PCR) and real-time PCR (qPCR) becoming the most prevalent techniques. Despite not yet being integrated into routine diagnostics, which are predominantly based on the Knott’s test and serological methods, these techniques offer significant benefits in the context of scientific research. This article proceeds to examine the potential of advanced techniques, such as high-resolution melting qPCR (HRM-qPCR), loop-mediated isothermal amplification (LAMP), droplet digital PCR (ddPCR), and microRNA (miRNA) detection, which are capable of enhanced sensitivity and early detection. The following work provides an in-depth analysis of the various diagnostic methods, emphasising the necessity of the continuous improvement and adaptation of these tools to effectively combat *D. immitis*. The findings underscore the importance of integrating these advanced methods into routine practice to improve detection rates and outcomes for infected animals.

## 1. Introduction

Dirofilariosis is a globally widespread vector-borne disease with zoonotic potential [1]. It is caused by nematodes of the genus *Dirofilaria*, which belong to the family Onchocercidae [2]. These nematodes have been detected in various mammalian species, predominantly in dogs [3,4,5,6], and less frequently in cats [7,8] or humans [9,10,11,12]. Approximately 40 recognised species of *Dirofilaria* have been identified [13], and at least six of them have been implicated in causing human infections: *Dirofilaria immitis*, *Dirofilaria repens*, *Dirofilaria striata*, *Dirofilaria tenuis*, *Dirofilaria ursi,* and *Dirofilaria spectans* [14]. Dirofilariosis in companion animals and humans is mainly caused by two nematode species, *D. immitis* and *D. repens* [15].

Despite being less frequently reported in human cases than *D. repens*, *D. immitis,* nevertheless, retains significant zoonotic importance due to its global occurrence. Furthermore, it is of paramount importance from both the veterinary and medical perspectives [2,14,16]. *D. immitis* is transmitted via blood-sucking mosquitoes belonging to the genera *Culex*, *Aedes*, *Ochlerotatus*, *Anopheles,* and *Mansonia* [17]. The domestic dog and certain wild canids are the typical definitive hosts for *D. immitis*, thereby acting as the primary reservoir for infection. Humans serve as the accidental dead-end host for *D. immitis* [18].

A definitive diagnosis of *D. immitis* infection in dogs can be challenging due to the non-specific nature of the associated symptoms. Such infections may range from asymptomatic to severe, with the potential to cause death [19]. It is well documented that *D. immitis* can cause symptoms affecting the cardiovascular and respiratory systems. However, the majority of these infections are asymptomatic [20]. The following symptoms have been observed: cough [21], increase in body temperature, abnormal heart and lung sounds [22], respiratory failure, dyspnea [21], activity intolerance, syncope [23], hepatomegaly, epistaxis [24], ascites [25], and decreased appetite and weight loss [26]. Table 1 delineates the clinical manifestations of *D. immitis* infection in canines, classified according to the level of severity.

*D. immitis* infection in humans can manifest with a range of clinical symptoms, including subcutaneous nodules, chest pain, cough, haemoptysis, wheezing, low-grade fever, and malaise [28]. Pulmonary dirofilariasis, caused by *D. immitis*, is typically asymptomatic; however, it may present with coin lesions on the lung, which can mimic malignancy [29]. In some instances, patients may present with an acute inflammatory response in the form of pneumonitis, caused by the pulmonary arterial occlusion of pre-adult worms, leading to pulmonary infarction and subsequent inflammation [30].

**Figure 1:**
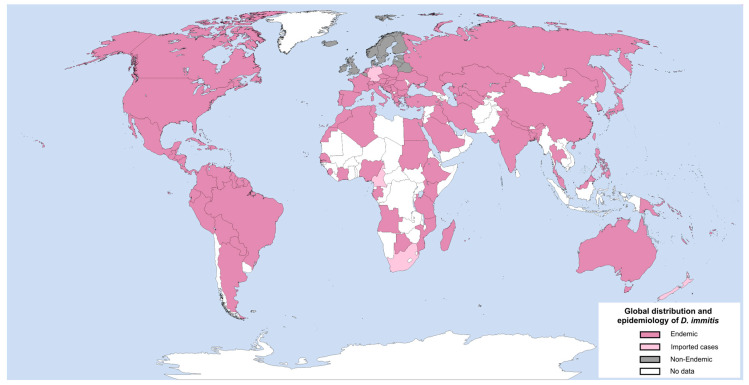
Global distribution and epidemiology of *D. immitis* (information was taken from Dantas-Torres et al. [46]). Created with GIMP 2.10.

*D. immitis* is a cosmopolitan species found in a variety of climates, including both coastal regions and inland continental areas. The prevalence of *D. immitis* varies widely across different geographical regions [31,32]. From an epidemiological perspective, the most recent data indicate a stable or even declining prevalence of *D. immitis* in Western European countries [33]. Furthermore, in Slovakia, it has become the predominant causal agent of dirofilariosis in the former endemic areas of *D. repens* distribution, consequently elevating the risk for both canines and humans [34]. In Russia, the highest incidence is observed in the southern, central, and eastern regions of the country with rates ranging from 36% to 55% [35,36]. Notwithstanding the aforementioned evidence, there are still new reports of *D. immitis* infections in canines [37,38,39], humans [40,41], and animals not previously considered as potential hosts [42]. The geographical range of *D. immitis* is expanding as a consequence of alterations in climatic conditions. As temperatures rise and weather patterns shift, the habitats of the mosquito vectors that transmit *D. immitis* are expanding, leading to an increase in the incidence of heartworm disease cases in previously unaffected areas [43]. Additional contributing factors include an underestimation of the risk, misdiagnosis (which is not always in accordance with international guidelines, even in endemic areas [44]), the lack of prophylaxis due to the unavailability of pharmaceuticals or financial constraints, and the high prevalence of untreated stray dogs and wild hosts [45]. Figure 1 illustrates the global prevalence of *D. immitis*, delineating endemic and non-endemic countries.

In accordance with the pervasive and geographically diverse distribution of *D. immitis* worldwide, the potential for severe clinical manifestations, and the confirmed zoonotic potential of the parasite, there is a pressing need for comprehensive research to identify the most effective diagnostic tests. This will facilitate the rapid, specific, sensitive, low-cost, and straightforward diagnosis of the infection, which will result in enhanced epidemiological control and a reduction in the spread of the parasite. This article, therefore, focuses on the diagnostic methods currently in use and potential techniques to improve the detection of *D. immitis*. This will enable the scientific community to identify research gaps and develop strategies for advancing the diagnostic techniques specific to *D. immitis* infection.

## 2. Methods for the Diagnosis of *D. immitis* Infection

The introduction of macrocyclic lactones into the pharmaceutical market has had a profound impact on the prevention of parasitic diseases, leading to a significant decline in investment in *D. immitis* research [47]. These drugs have been demonstrated to be highly efficacious in the prevention of canine dirofilariosis, particularly by controlling the third (L3) and fourth (L4) larval stages of the parasite [48]. The prevention of *D. immitis* infection in dogs represents a safer and more proactive strategy than the removal of adult parasites [18,49]. A significant challenge in the control and eradication of infections is posed by the emergence of *D. immitis* parasites exhibiting resistance to macrocyclic lactones [50,51,52,53,54]. The routine use of antiparasitic agents as preventive measures, when they are ineffective against resistant genotypes, are associated with a high probability of the further spread of resistance and an increase in infection rates, which may pose a serious public health risk [55]. Notwithstanding the ongoing prevalence and significant impact of parasitic diseases on human health and animal welfare, research in this field constitutes a relatively minor percentage of total funding. In 2020, grants allocated for research on communicable, maternal, and perinatal conditions, including parasitic diseases, made up only 26.3% of the total global funding for biomedical research [56]. Moreover, conducting experiments with parasites presents inherent challenges, particularly when it comes to nematodes such as *D. immitis*, whose life cycle encompasses both arthropods and mammals. This contributes to the financial and logistical complexity of maintaining the optimal parasite population for the purposes of the study. This is particularly problematic in the case of *D. immitis*, as dogs are the preferred definitive host of the parasite, and research on them is both very costly and fraught with ethical and legal conflicts [57]. The following sections present an overview of the current state of knowledge and advances in basic research on methods to develop effective diagnostic strategies in the context of *D. immitis* infection, as well as the prospects for future innovations in this field.

### 2.1. Microscopy-Based Diagnostic Methods

The diagnosis of parasitic infections is dependent on a series of laboratory techniques which are employed in order to detect organisms in clinical samples through their morphological characteristics and visual identification [58]. The morphological traits of various species are complex and subtle, thereby limiting the number of diagnostic procedures that can be automated [59]. The ability to utilise a microscope for the analysis of parasite morphology is dependent on extensive training, with the accuracy of identification being contingent upon the examiner’s expertise [60]. In order for parasitological diagnostics to be effective and accurate, laboratory professionals must possess a comprehensive understanding of the life cycles, epidemiology, invasiveness, geographic distribution, clinical manifestations, and recommended treatments of the parasites [61].

The microscopy-based diagnosis of *D. immitis* infection is primarily based on the identification of circulating microfilariae in the whole blood through the microscopic examination of capillary peripheral blood smears [62] and their concentration using the modified Knott test [63,64] or filter test [65]. The principal diagnostic characteristics pertain to the variations in the morphology and dimensions of the different structures of the parasite [1]. The modified Knott test is regarded as the gold standard for conducting microfilarial examinations and is the preferred method for morphometric analyses [66]. The traditional microscopy-based methods, which are considered the gold standard for the diagnosis of infections [67], have a number of inherent disadvantages and problems when used for the detection of *D. immitis*. In areas where the prevalence of nematode infection is high, it has been observed that approximately 20% to 39% of dogs infected with *D. immitis* may not have circulating microfilariae [68]. This is primarily attributable to the prior administration of microfilaricidal antiparasitic pharmaceuticals, which can result in the absence of circulating microfilariae even in dogs infected with *D. immitis* [18]. Additionally, the low sensitivity of the tests may also be attributed to the presence of same-sex parasite infections, low parasite titres in the host, or host immune responses against adult nematodes or microfilariae [1]. Furthermore, the subperiodic nature of microfilariae occurrence in the host bloodstream [69], whereby microfilariae of *D. immitis* are always present but in variable concentrations, has a significant impact on the accuracy of diagnosis when the timing of biological sampling for analysis is not optimal [70]. Furthermore, the lack of an efficient method for testing a large number of samples in a short period of time represents a significant challenge [71]. Although the identification of parasites can be achieved through the assessment of cephalic and caudal morphology, the differentiation between nematode species based on these characteristics can often prove to be problematic, potentially complicating the process of reaching a definitive diagnosis [15]. Furthermore, the identification of multiple nematode species in animals co-infected with multiple parasites represents a significant challenge [64]. Consequently, the sensitivity of tests based on the detection of microfilariae is frequently inadequate for the purpose of excluding infection in the case of a negative result and for identifying specific nematode species. It is, therefore, necessary to employ supplementary diagnostic techniques, such as antigenic tests and molecular methods, in order to facilitate a more accurate identification and exclusion of infection. Table 2 presents a comparative analysis of the advantages and disadvantages of the various microscopy-based methods employed in the diagnosis of *D. immitis* infection.

The morphological identification of parasites has constituted the foundation of parasitic disease diagnosis for centuries. Although it remains a valuable diagnostic tool, there is a need to develop new diagnostic methods that are highly specific and sensitive, easy to perform, and cost-effective, and can be applied on a large scale in diverse laboratory and field settings. Promising avenues for development include molecular methods that allow the rapid and precise amplification of DNA or RNA from different parasite species, immunoenzymatic methods to detect antigens or antibodies, and proteomic and transcriptomic studies to identify specific proteins that may serve as specific markers for the presence of *D. immitis* in the host.

### 2.2. Serological Tests

The advent of serology-based diagnostic techniques has marked a profound transformation in the diagnosis of parasite infections. These tools have the potential to facilitate routine screening of asymptomatic dogs and confirm infection in dogs exhibiting clinical signs [74,75]. Such tests are categorised into two principal types: antigen-detection tests and antibody-detection tests [76]. Examples of these include ELISA and immunochromatographic rapid diagnostic tests. In order to detect the presence of the *D. immitis*, serological tests are based on the detection of specific circulating antigens in a sample of whole blood, plasma, or serum [27]. Serological tests, whether employed as a standalone technique or in conjunction with other methods, represent the most frequently utilised approach for the diagnosis of nematode infections in dogs and cats [44]. Such tests are commercially available and commonly used in veterinary clinics for the detection of *D. immitis* infections [2]. Examples of commercially available ELISA tests include the following:

- DiroCHEK^®^ (Zoetis, Parsippany, NJ, USA);
- PetChek^®^ HTWM PF (IDEXX Laboratories, Westbrook, ME, USA).

Examples of commercial immunochromatographic rapid diagnostic tests are as follows:

- VETSCAN^®^ Heartworm Rapid Test (Abaxis, Union City, CA, USA);
- SNAP 4Dx Plus Test (IDEXX Laboratories, Westbrook, ME, USA);
- Anigen Rapid CHW Ag Test Kit 2.0 (Bionote, Hwaseong, Republic of Korea);
- WITNESS^®^ Heartworm Rapid Test (Zoetis, Parsippany, NJ, USA);
- Solo Step^®^ CH (Heska, Loveland, CO, USA).

The manufacturers of the aforementioned tests indicate very high sensitivity and specificity on their respective websites, with reported results ranging from 94% to 100% for both parameters. In contrast, the results of published studies examining the sensitivity and specificity of antigen tests for the detection of *D. immitis* demonstrate notable inconsistencies. In the majority of cases, the aforementioned parameters are found to be lower than those claimed by manufacturers. Furthermore, significant differences are observed between the various test kits utilised [62,77,78,79].

Despite the manufacturer’s assurances of high specificity and sensitivity, they are not always reliable for the detection of *D. immitis* infection. It is possible for false-negative results to occur in cases of low parasite titres and low antigenemia, as well as in instances where only adult male nematodes are present [2]. Furthermore, the sensitivity of these methods is reduced when adult female nematodes are absent [77], or when the host has a very low number of female parasites. Such results are a consequence of the utilisation of monoclonal antibodies in the tests, which predominantly detect female-specific antigens. Moreover, the correlation between the amount of *D. immitis* antigen in the blood and the number of adult female parasites is not always accurately reflected in these tests [80]. The presence of antigen–antibody complexes in the host serum has the potential to interfere with and mask the reactivity of the antigen, which is associated with a reduction in the test sensitivity and the occurrence of false-negative results [81,82,83]. It has been established that heat treatment of serum samples prior to testing enhances test accuracy by facilitating antigen release and the breakdown of immune complexes [84,85]. It is not currently recommended that blood samples be subjected to routine heating, and this procedure is not included in the instructions for any commercially available antigen test for *D. immitis*. Another potential cause of false-positive results is cross-reactivity with other parasite antigens that may react in some test kits. The antigens may have originated from parasites such as *D. repens* [86], *Angiostrongylus vasorum* [87], *Spirocerca lupi* [88], and *Acanthocheilonema dracunculoides* [89].

In light of the inherent limitations of serological tests for the detection of infection due to *D. immitis*, it is imperative that the results be identified only as ‘positive’ or ‘no antigen detected’. It is inadvisable to employ the term ‘negative’ in this context, as this might be interpreted to imply a complete absence of infection, which is not always a definitive conclusion. The interpretation of antigen test results should be based on the integration of pertinent clinical data, including symptoms, and the utilisation of supplementary testing methodologies, such as molecular techniques, is imperative to confirm or refute the presence of an infection.

### 2.3. Molecular Methods for Diagnosis of Infection

The advent of molecular biology and its subsequent rapid development for use in parasitic diagnostics constituted a significant advancement. This has provided diagnosticians with an invaluable ability to simultaneously detect and identify different parasitic organisms not only in clinical samples but also in their natural vectors [90]. It is crucial to develop novel molecular diagnostic techniques that allow the precise and sensitive detection of parasites within the host. The following sections thus set out to describe potential methods for the molecular diagnosis of *D. immitis* infection.

#### 2.3.1. Polymerase Chain Reaction (PCR)

PCR-based diagnostic methods, which focus on DNA amplification and sequencing, have high specificity and can accurately differentiate between parasites of the same genus, such as *D. repens* and *D. immitis*. The molecular detection of *D. immitis* has been demonstrated to be a highly sensitive, specific, and reliable method for the detection of small quantities of genomic DNA in the blood of dogs [62] and even in vectors such as mosquitoes [91].

In a study conducted by Oh et al. (2017), *D. immitis* cytochrome c oxidase subunit I (*COX1*) was amplified from genomic DNA isolated from the peripheral blood of infected dogs. The *COX1* genes of seven filarial nematodes were compared: *Setaria tundra*, *Setaria digitata*, *D. repens*, *B. malayi*, *W. bancrofti*, and *O. volvulus* [92]. This gene is described in the literature as a ‘barcode gene’ for filarial nematodes [93]. Due to the high copy number relative to the nuclear gene in each cell and the high amplification efficiency, even minimal amounts of DNA can be detected [94]. A highly conserved region of the *COX1* gene of *D. immitis* was identified from the collation, thereby enabling the amplification of a 150 bp long fragment. The identification of the *COX1*-specific region of *D. immitis* enabled its amplification with designed primers, offering the potential for the highly specific and sensitive molecular diagnosis of infection caused by the parasite. This methodology was employed in a study carried out by Soares et al. (2022). The developed PCR served as a reference test for the comparison of the specificity and sensitivity of infection detection by microscopy-based methods (capillary blood smear, peripheral blood smear, and modified Knott test) and a serological technique (rapid test kit—Dirofilariose AG Test kit^®^, Alere, Waltham, MA, USA). This enabled the demonstration that all of the evaluated methods exhibited a high detection rate of infection [62].

#### 2.3.2. Real-Time PCR (qPCR)

One approach to detecting *D. immitis* is to perform a quantitative analysis of microfilarial DNA content in the blood of dogs and mosquitoes [95,96]. These tests are commonly used for population screening, with the objective of detecting *D. immitis* [97,98,99,100,101]. Multiplex qPCRs are the most commonly used form of qPCR, as they facilitate the simultaneous amplification and detection of multiple target genes [102]. Multiplex qPCR uses fluorophore probes with distinct emission wavelengths. Subsequently, the fluorescent signals are then separated and detected using different optical channels. In theory, the number of target genes for simultaneous detection is unlimited, provided that the emission wavelengths of the different probes do not overlap [103].

In 2020, Laidoudi et al. developed a technique for the simultaneous detection of *D. immitis*, *D. repens*, and *A. reconditum* as well as for latent *Dirofilaria* spp. infections in dogs. The method was further enhanced by the incorporation of a duplex qPCR assay for the detection of *D. immitis* and *A. vasorum*, which enabled the differentiation of heartworms in dogs. The triplex TaqMan qPCR method amplified a 166 bp fragment of the *COX1* gene and its specificity was based on the use of TaqMan probes and dedicated dyes. The duplex TaqMan qPCR method for *D. immitis* and *A. vasorum* detected a 227 bp fragment of the *COX1* gene. The protocol developed allowed the detection of up to 1.5 × 10^−4^ microfilariae per ml and demonstrated an efficiency of 99.1 to 100% for all species tested [104].

The quantification of microfilariae is of significant importance for the monitoring of treatment progress and the detection of resistance to macrocyclic lactones. In a study published in 2024, Lau and colleagues compared the efficacy of a modified Knott test with that of a qPCR targeting the DNA of *D. immitis* and associated *Wolbachia* endosymbiont in canine blood samples [105]. For this purpose, genomic DNA samples were evaluated using a TaqMan qPCR assay targeting *D. immitis*, *Wolbachia* spp., and *Canis lupus familiaris* DNA. The qPCR assay demonstrated high efficiency, with a value of 90–100% and a correlation coefficient greater than 0.94. Considering efficiency and speed, the TaqMan qPCR assay is a suitable alternative to the modified Knott assay for the quantification of microfilariae (Cohen’s kappa coefficient [κ]: κ = 1 using the qPCR marker *D. immitis*, κ = 0.93 using the qPCR marker *Wolbachia*) [105]. The results of the qPCR analysis were found to be comparable to those of the quantitative modified Knott test of *D. immitis* in dogs before and after the administration of macrocyclic lactone [106].

qPCR provides an alternative for the diagnosis and monitoring of dirofilariosis, including the quantification of parasite titres. An increasing number of studies conclude that the qPCR method is a viable option for the detection of mf in parallel with antigen tests for canine heartworm infection [107]. The incorporation of molecular tests in the diagnosis of infection provides an effective alternative to diagnostic screening for *D. immitis*.

#### 2.3.3. High-Resolution Melting qPCR (HRM-qPCR)

In recent years, HRM-qPCR has emerged as a prominent approach in parasitological diagnostics [108,109,110]. The technique involves denaturing PCR products in real time. Fluorescence changes resulting from the release of an intercalating dye from the DNA strand are monitored. The analysis of melting curves for unknown samples, in comparison with those of known isolates, allows for the identification of specific strains or species [111].

In a study conducted by Wongkamchai et al. (2013), the HRM-qPCR assay targeted the mitochondrial 12S rRNA gene, which is highly conserved and contains genus and species-specific sequence variations useful for identifying three species of filaria, *Brugia pahangi*, *B*. *malayi*, and *D*. *immitis*, in blood samples from cats and dogs. The HRM-qPCR developed by the authors could detect 1 microfilariae/reaction or 1 microfilariae/60 μL of blood sample. Moreover, DNA sequencing of the samples tested showed that they were 100% identical to the HRM-qPCR result, thus confirming that it is as effective as sequencing. Furthermore, the HRM-qPCR technique enables the differentiation between *D. immitis* and *D*. *repens*. The differentiation of the two *Dirofilaria* species via microscopy-based methods can prove challenging and may result in misdiagnosis, particularly in samples from regions where both species are present [112]. Albonico et al. (2014) designed a rapid and cost-effective HRM-qPCR protocol for the simultaneous and unambiguous detection and differentiation of the microfilarial DNA of *D. immitis* and *D. repens* extracted from canine peripheral blood samples. The method does not necessitate the utilisation of probes and multiplex methods or DNA sequencing for the expeditious detection of two closely related *Dirofilaria* species. The efficiency and costs of HRM-qPCR were comparable or cheaper than those of PCR and sequencing, with a very rapid detection step following amplification [113]. Rojas et al. (2015) conducted an evaluation of an assay based on HRM-qPCR for the detection of filaria in dogs and a comparison of its performance with other diagnostic techniques. The presence of nematodes in blood samples was investigated using four different methodologies: microhaematocrit test (MCT), modified Knott’s test, serological analysis, and HRM-qPCR. The HRM-qPCR method demonstrated a 94.3% detection rate for positive results, while the MCT, Knott’s test, and serology exhibited detection rates of 37.1%, 71.4%, and 45.7%, respectively [114].

The high sensitivity and specificity of HRM-qPCR have led to its significant recognition in the field of molecular diagnostics. Its capacity to accurately differentiate between DNA sequences renders it a promising tool for the screening and mapping of *D. immitis* infections [115,116].

#### 2.3.4. Loop-Mediated Isothermal Amplification (LAMP)

The LAMP method, initially described in 2000 [117], represents a straightforward alternative to PCR for colorimetric reading [118]. It is particularly beneficial in resource-limited settings where the costly equipment necessary for PCR is unavailable. Furthermore, LAMP is also less susceptible to the inhibitory effects of compounds present in clinical samples and mosquitoes, which can impede the activity of *Taq* polymerase in PCR [119]. This technique involves synthesis using *Bst* DNA polymerase, which functions at isothermal temperatures without denaturing the DNA template [120]. This allows the amplification of the template up to 10^9^–10^10^ times within a period of 15–60 min. The results are read by visual inspection of the turbidity induced by the precipitation of white magnesium pyrophosphate or by UV inspection using a fluorescent dye [121]. The potential of LAMP as a diagnostic test for neglected tropical diseases (NTDs) has been a subject of intensive investigation from the outset. The first LAMP test for the detection of human African trypanosome DNA was published as early as 2003, marking the advent of a new era in NTD diagnosis [122]. Subsequently, LAMP has been acknowledged as a potential alternative to PCR-based methods [58]. Similar to PCR and qPCR, the LAMP-based assay employs the amplification of the *COX1* gene. In 2009, Aonuma et al. developed a rapid, simple, and highly sensitive method for testing vectors carrying zoonotic pathogens including *D. immitis*. The method allowed for the detection of up to one individual of *D. immitis* that would have been undetected by conventional microscopic analysis. Moreover, the successful detection of *D. immitis* in mosquitoes demonstrated the applicability of this method in field studies [123].

The LAMP method demonstrates considerable promise as a diagnostic tool, particularly in the context of NTDs and field studies. The simplicity, low hardware requirements, and high sensitivity, even with low parasite counts, makes it a promising alternative to classical PCR methods. Nevertheless, further studies and validations are required to fully assess the efficacy and reliability of the method under different conditions.

#### 2.3.5. Droplet Digital PCR (ddPCR)

ddPCR is a widely used method in the field of parasitology for the detection and quantification of various pathogens [124,125,126]. The technique allows for the absolute quantification of parasite DNA without the need for a reference curve, resulting in highly accurate and reproducible measurements [127]. ddPCR involves dividing the sample into tens of thousands of water-in-oil nanodroplets within a single sample of genomic DNA and a primer–probe pair undergoing simultaneous PCR amplification and quantification of the amplicon [128]. This method is more sensitive than qPCR at lower parasite DNA concentrations, reliably detecting low parasite titres and providing higher sensitivity in diagnosis [129]. The technology offers distinct advantages over qPCR, including an enhanced resilience to inhibitors and the capacity for absolute quantification without the necessity of a standard curve. This renders it a promising option for parasite detection and quantitative analysis [130].

The ddPCR method is proving useful for the detection of macrocyclic lactones resistant *D. immitis*. Kumar et al. (2023) developed a protocol based on ddPCR for the detection of single nucleotide polymorphisms (SNPs) correlated with resistance to macrocyclic lactones. They assessed the frequencies of resistance-associated alleles in *D. immitis* populations and compared the results obtained with MiSeq sequencing data. The developed method distinguished four distinct clusters to be distinguished allowing for the simultaneous detection and quantification of target SNP wild-type and mutant alleles. The ddPCR assay accurately detected and quantified alternative nucleotides in two reference isolates, ML susceptible Missouri (MO) and ML resistant JYD-34, at previously identified SNP positions [131].

Despite its numerous advantages and high diagnostic potential, ddPCR is still rarely used. However, its ability to absolutely quantify and detect low numbers of parasites in the host makes it an extremely valuable diagnostic tool [126,132,133].

### 2.4. miRNA Detection

In recent years, the detection of circulating DNA and microRNAs (miRNAs), and their role in the infection, has been a crucial area of research within all nematode parasites [134]. miRNAs in *D. immitis* have emerged as key regulators of gene expression, with significant implications for parasite biology and potential treatments for dirofilariasis. Research has shown that miRNAs, such as miR-34 and miR-71, which are specific to filarial nematodes, can serve as reliable biomarkers for detecting the presence of *D. immitis* infections in dogs, although they do not provide information on the intensity of the infection [135]. This discovery highlights the potential role of these miRNAs in discriminating between infected and uninfected hosts, indicating their importance in regulating gene expression within the parasite.

miRNAs in *D. immitis* are integral to the regulation of gene expression, which significantly influences the pathogenicity and virulence of the parasite. Furthermore, the species specificity and evolutionary implications of miRNAs in *D. immitis* and related nematodes provide fascinating insights into the complex interactions between these parasites and their hosts, potentially guiding future research and therapeutic strategies.

## 3. Conclusions

The detection of *D. immitis* is an ongoing process that is evolving in response to the parasite’s widespread distribution, severe clinical impact, and potential for transmission between animals and humans. The implementation of accurate, efficient, and cost-effective diagnostic methods is crucial for enhancing epidemiological control and curbing the dissemination of *D. immitis*. While traditional morphological identification remains a valuable approach, it is necessary to complement this with newer diagnostic techniques that offer greater specificity, sensitivity, and ease of use. The recent advancements in molecular diagnostics have significantly enhanced our ability to detect *D. immitis* with high precision. Techniques such as qPCR, HRM-qPCR, and LAMP provide robust alternatives to conventional methods, offering rapid and reliable results even at low parasite concentrations. These methods facilitate not only early detection but also improve the accuracy of diagnosing infections in comparison to traditional serological tests. Molecular techniques, including ddPCR, offer the capacity to quantify parasite DNA with exceptional sensitivity, rendering them valuable for the assessment of low-level infections and the characterisation of resistance profiles. Similarly, the investigation of miRNAs and their function in the biology of *D. immitis* has the potential to facilitate the development of new diagnostic and therapeutic strategies. While these advancements represent a significant step forward, it is essential to address the current limitations of current diagnostic methods. The validation of newer molecular techniques remains incomplete, particularly with regard to their application in single adult worm infections or microfilariae-negative (MF-negative) cases, which are critical for assessing the risk of treatment and prophylaxis. While these methods are more sensitive and specific in identifying microfilariae-positive (MF-positive) infections, they do not yet fully address the challenge of detecting MF-negative infections, which represents a significant gap in diagnostic accuracy. The aforementioned limitation is of particular consequence when considering the treatment risks often associated with MF-negative infections, which necessitate the implementation of targeted intervention measures. Therefore, future research must prioritise the refinement and validation of these newer diagnostic tools to ensure their efficacy in detecting both MF-positive and MF-negative cases across diverse clinical settings. By addressing these gaps, it is possible to advance our diagnostic capabilities and improve both the management of *D. immitis* infections and the outcomes for both veterinary and public health. In light of these advancements, it is of the utmost importance to continue refining and validating these diagnostic approaches in order to ensure their efficacy across diverse settings. The integration of molecular diagnostics with clinical assessments will enhance the overall accuracy of *D. immitis* detection, thereby offering improved outcomes for both veterinary and public health. Future research should focus on addressing existing gaps in diagnostic methodologies and improving the accessibility of advanced diagnostic tools. By advancing our diagnostic capabilities, we can better manage *D. immitis* infections and mitigate their impact on both animal and human health.

## Figures and Tables

**Table 1:** The clinical signs of a *D*. *immitis* infection in canines (information was taken from the American Heartworm Society [27]).

Degree of Severity	Symptoms
Mild	Asymptomatic or cough
Moderate	Cough, activity intolerance, abnormal lung sounds
Severe	Cough, activity intolerance, dyspnea, abnormal heart and lung sounds, hepatomegaly, syncope, ascites, death

**Table 2:** Summary of the advantages and disadvantages of the microscopy-based methods employed in the diagnosis of *D. immitis* infection (information was taken from Genchi et al.; ESDA [72,73]).

Microscopy-Based Methods	Advantages	Disadvantages
Blood smears from peripheral veins and capillaries	Not time-consuming Straightforward and uncomplicated Inexpensive	Low sensitivity High rate of false-negative results Unable to distinguish between species of microfilariae
Modified Knott test	Capability to differentiate microfilariae belonging to different species	Time-consuming Requires a good knowledge of microfilariae morphology
Filter test		Expensive test kits (Difil-Test^®^, Vetoquinol USA, Fort Worth, TX, USA) Lysate solution may cause diminishment of microfilariae

## Data Availability

Not applicable.
